# Resilience among clinical pharmacists and related factors: a cross-sectional study

**DOI:** 10.1186/s40780-025-00498-3

**Published:** 2025-10-24

**Authors:** Yusuke Tsuchiya, Wataru Arai, Nobuko Sunami, Hiroyuki Hosono, Tae Maeshima, Fumio Itagaki, Machiko Watanabe

**Affiliations:** 1 Department of Pharmacy, Ageo Central General Hospital, 1-10-10, Kashiwaza, Ageo-shi, Saitama, 362-8588 Japan; 2 Faculty of Pharmaceutical Science, Teikyo University, 2-11-1, Kaga, Itabashi-ku, Tokyo, 173-8605 Japan; 3 Faculty of Medical Technology, Teikyo University, 2-11-1, Kaga, Itabashi-ku, Tokyo, 173-8605 Japan; 4 Graduate School of Pharmaceutical Sciences, The University of Tokyo, 7-3-1 Hongo, Bunkyo-ku, Tokyo , 113-0033 Japan

**Keywords:** Resilience, Clinical pharmacist, Self-efficacy, Burnout, Bidimensional resilience scale, Innate resilience, Acquired resilience

## Abstract

**Background:**

Resilience has recently attracted attention as a means of coping with challenging situations. Although there have been several studies on resilience among healthcare professionals, there are limited reports on resilience among pharmacists. In this study, we conducted a survey of resilience among clinical pharmacists and examined factors related to self-efficacy, burnout, and work.

**Methods:**

Clinical pharmacists at 38 medical institutions were surveyed regarding basic attributes, work status, resilience, self-efficacy, and burnout using a web-based questionnaire. Descriptive statistics for each survey item were calculated, and exploratory factor analysis was conducted. The relationships between resilience scores and each factor were examined using Spearman’s rank correlation coefficient (ρ), the Mann–Whitney *U* test, and the Kruskal–Wallis test. A multiple regression analysis was conducted using resilience scores as the objective variable and other factors as explanatory variables. The “Bidimensional Resilience Scale” was used to measure resilience.

**Results:**

Responses were obtained from 285 participants, which confirmed the reliability of the psychological scale. Factor analysis extracted five new factor structures but confirmed that the two-dimensional structure was maintained. The correlations were significant for self-efficacy, burnout, and the percentages of research, teaching, and other work (RTOW). Multiple regression analysis suggested that “self-efficacy” was the factor most strongly associated with resilience (overall), innate resilience, and acquired resilience.

**Conclusions:**

This study revealed the relationship between resilience, self-efficacy, and RTOW among clinical pharmacists in Japan. Criterion-related validity was also evidenced by high self-efficacy. RTOW being newly identified as an associated factor in this context provides insights for further development of the scale.

## Background

In Japan, owing to the emergence of an aging society with a low birth rate, medical care is becoming increasingly sophisticated and diversified. In this context, expectations regarding pharmacists’ social role are increasing, and there is a need to improve the quality of pharmacists [1]. Simultaneously, pharmacists need to be trained in practical skills to respond appropriately in clinical settings [2].

Resilience, a concept referring to the psychological ability to cope with challenging situations, has attracted attention in recent years. The attributes that define resilience include rebounding, determination, self-efficacy, and social support [3]. In the field of nursing, several studies on resilience have been conducted [4–6]. Resilience is considered a useful indicator of the ability to overcome challenges, and scales to measure it are being developed [7]. Reporting on the relationship between resilience and the ability to practice nursing, Tomita et al. stated that support to enhance resilience is necessary for nurses to continue working and improve nursing care quality [8].

The American College of Clinical Pharmacy (ACCP) defines a clinical pharmacist as a professional who provides care in a multidisciplinary team by optimizing drug therapy for individual patients [9]. According to a study conducted by Jon et al. [10], pharmacists often experience job stress, burnout, and moral distress, while well-being and resilience play a crucial role in preparing individuals to thrive. Weiss et al. reported that individuals with higher resilience levels experience lower rates of burnout and improved occupational performance [11].

In Japan, as medical care becomes increasingly sophisticated, workload increases, interpersonal work increases, and a growing number of clinical pharmacists are placed in mentally and physically challenging situations. Therefore, the development of resilience and the establishment of support systems for clinical pharmacists must be considered. However, research on resilience among pharmacists in Japan is limited, and the actual situation is yet to be clarified.

In this study, we investigated resilience among clinical pharmacists and identified factors related to self-efficacy [8] and burnout [11], which are related to resilience and the work of clinical pharmacists.

## Methods

### Participants

#### Target population and data collection method

This cross-sectional study used a web-based questionnaire targeting clinical pharmacists working in hospitals. In this study, clinical pharmacists were defined as pharmacists who had acquired the qualities expected of pharmacists in the field of medicine and were involved in the pharmacotherapy of patients in collaboration with multiple professionals based on their pharmacological knowledge.

In Japan, the practice by which pharmacists contribute to patient pharmacotherapy through collaboration with other healthcare professionals is referred to as “ward pharmacy services.” Accordingly, for this study, clinical pharmacists were regarded as pharmacists working in hospitals where such services are provided, irrespective of their years of experience, qualifications, or job titles. To minimize variability in professional background, community pharmacists and those working in institutions without ward pharmacy services were excluded. In hospitals with ward pharmacy services, even pharmacists not directly assigned to a ward are generally engaged in multidisciplinary activities such as reviewing prescriptions, responding to queries, handling consultations from other professionals, and participating in meetings or educational sessions. Therefore, all pharmacists working in such hospitals were included as clinical pharmacists for the purposes of this study.

The questionnaire was sent via e-mail to the pharmacy department administrators of 38 medical institutions with 20 or more beds in Tokyo and nine prefectures in the Kanto Koshinetsu region, requesting their cooperation in the survey. A responsible individual at each institution distributed the questionnaire to the participants, and individuals who volunteered to participate accessed the web-based questionnaire to answer the questions. The data were subsequently collected by the information manager, who was a member of the research team.

#### Contents of the questionnaire

The questionnaire included 21 items from the resilience scale, 23 items from the self-efficacy scale, and 17 items from the burnout scale, in addition to questions on basic attributes (gender, age, years of experience as a pharmacist, years of experience at the hospital, and classification of the facility where the pharmacist works) and the work status of clinical pharmacists (percentage of work type performed, number of patient guidance sessions on medications, number of medication instructions, number of prescribing suggestions, number of PREAVOID cases, number of conference presentations, and number of scientific papers submitted). The Percentage of Work type performed was calculated as the sum of four types of work: clinical practice work (CPW); dispensing room work (DRW); managerial work (MW); and research, teaching, and other work (RTOW). The percentage of work was surveyed such that the total percentage of work was 100%. The term “PREAVOID” was coined by the Japan Hospital Pharmacists Association based on “PREvent and AVOID” adverse drug reactions. PREAVOID refers to cases in which pharmacists are directly involved in drug therapy to avoid or reduce patient disadvantages (adverse drug reactions, drug interactions, and inadequate therapeutic effects) through the practice of pharmacological patient care.

### Psychological scales

**Fig. 1:**
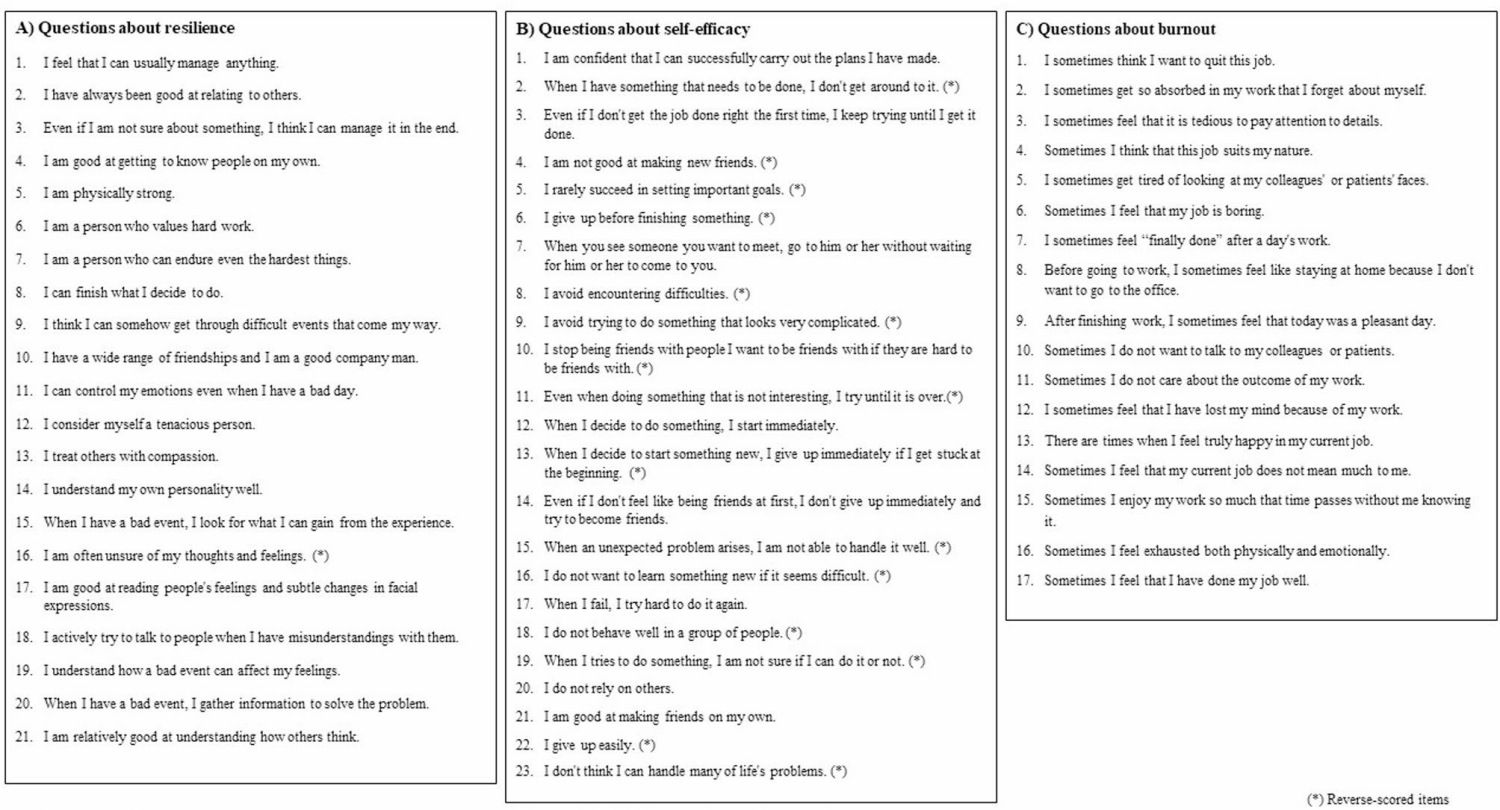
Psychological Scales and Question Contents. **A**) Resilience Scale. The resilience scale consists of 21 items (“1” to “12” are innate resilience items and “13” to “21” are acquired resilience items), each rated on a 5-point Likert scale (“1: not at all applicable,” “2: not applicable,” “3: undecided,” “4: moderately applicable,” and “5: highly applicable”). Total scores range from 21 to 105 (* indicates reverse-scored items; innate resilience: 12–60, acquired resilience: 9–45), with higher scores indicating greater resilience levels. **B**) Self-efficacy Scale. The self-efficacy scale consists of 23 items, each rated on a 5-point Likert scale (“1: not at all applicable,” “2: not applicable,” “3: undecided,” “4: moderately applicable,” “5: highly applicable”). Total scores range from 23 to 115 (* indicates reverse-scored items), with higher scores indicating greater self-efficacy levels. **C**) Burnout Scale. The burnout scale consists of 17 items, each rated a 5-point Likert scale (“1: never,” “2: rarely,” “3: sometimes,” “4: often,” “5: always”) over a recent 6-month period. Total scores range from 17 to 85, with higher scores indicating greater burnout levels

Three validated and reliable psychological scales were used in this study (see Fig. 1). Each was scored on a 5-point Likert scale, with total scores calculated for analysis.

#### Resilience

Resilience was measured using the Bidimensional Resilience Scale (BRS) developed by Hirano [7], which evaluates four innate factors (optimism, control, sociability, vitality) and three acquired factors (problem-solving, self-understanding, understanding others’ psychology). This distinction reflects differences in the influence of innate characteristics and the ease of intentional acquisition [12]. The reliability and validity of the BRS have been confirmed [7, 12], and the scale was deemed suitable for evaluating resilience in clinical pharmacists.

#### Self-efficacy

Self-efficacy was measured using the Japanese version of the Generalized Self-Efficacy Scale (JGSES) developed by Narita et al. [13]. Self-efficacy can be classified into two types: one that influences behavior in specific tasks or situations, and another that influences behavior across a wide range of everyday situations, independent of specific contexts [14]. The JGSES measures the latter—generalized self-efficacy—and was selected to assess self-efficacy irrespective of particular contexts.

#### Burnout

Burnout was evaluated using the Japanese Burnout Scale (JBS) developed by Kubo [15]. Adapted from the Maslach Burnout Inventory, the JBS was tailored to the Japanese human services field. Its reliability and validity have been established, and it is suitable for assessing burnout among clinical pharmacists, whose work is highly interpersonal in nature.

### Statistical analyses

#### Survey of actual resilience and exploratory factor analysis

Descriptive statistics were calculated for each survey item. To verify the reliability of the resilience scale, ceiling and floor effects were checked and Cronbach’s alpha coefficients were calculated from the existing factor structure. Exploratory factor analysis was conducted on the 21 questions to confirm the pharmacists’ unique factor structure.

#### Investigating factors associated with resilience

The relationships between resilience scores and each factor were examined using Spearman’s rank correlation coefficient (ρ) for ordinal variables, the Mann–Whitney *U* test for two-group comparisons, and the Kruskal–Wallis test for comparisons among more than two groups. Effect sizes were also calculated, with *r* for the Mann–Whitney *U* test and *η²*_*H*_ for the Kruskal–Wallis test. The Functional Classification of Hospitals was excluded from the survey because it was difficult to quantify. Gender was quantified as 0 for males and 1 for females. Continuous count data were not available for items such as the number of patient guidance sessions on medications, prescribing suggestions to physicians, PREAVOID cases, research presentations, and submitted academic papers, as the survey collected approximate counts for these variables. Consequently, they could not be regarded as continuous data with equal intervals and were treated as ordinal categorical data for statistical analysis. Self-efficacy and burnout were included in the analyses for criterion-related validity. Innate and Acquired Resilience were calculated using a factor structure newly identified in this study, rather than the original scale’s classification.

Multiple regression analysis was conducted using resilience scores as the objective variable and other factors as explanatory variables. A stepwise method was used for analysis.

The significance level was set at 5%, and a correlation coefficient (ρ) of 0.20 ≦ | ρ | < 0.40 was defined as weakly correlated, 0.40 ≦ | ρ | < 0.70 as correlated, and 0.70 ≦ | ρ | ≦ 1.0 as strongly correlated.

SPSS Statistics for Windows (Version 29.0.2.0. IMB Corp., Armonk, NY, USA) was used for the statistical analysis.

## Results

### Participants’ attributes

In total, 285 respondents were recruited from 38 cooperating facilities. Participants’ attributes are listed in Table 1.

**Table 1:** Participants’ attributes

Survey item	N=285
Gender †	
male	121 (42.5)
female	164 (57.5)
Age †	
20's	92 (32.3)
30's	105 (36.8)
40's	63 (22.1)
50's	22 (7.7)
60's	3 (1.1)
Functional Classification of Hospitals †	
Hospital with Specific Functions	72 (25.2)
General Hospitals: More than 500 beds	84 (29.5)
General Hospitals: 100–499 beds	110 (38.6)
General Hospitals: 20–99 beds	5 (1.7)
Chronic Care Hospitals	7 (2.5)
Convalescent Hospitals	7 (2.5)
Years of pharmacist experience (year)*	10 (4–16)
Years of hospital experience (year)*	9 (3–16)
Percentage of work in the past year (%) *	
Clinical Practice Work (CPW)	50 (20–66.7)
Dispensing Room Work (DRW)	30 (15–50)
Managerial Work (MW)	0 (0–10)
Research, Teaching, and Other Work (RTOW)	5 (0–10)
Number of patient guidance sessions on medications in the past year †	
Less than 10 cases	52 (18.2)
10-49 cases (about 2-3 cases per month)	14 (4.9)
50-99 cases (about 5 cases per month)	14 (4.9)
100-299 cases (about 5-10 cases per month)	23 (8.1)
300-599 cases (about 10-50 cases per month)	77 (27.0)
600-1199 cases (about 50-100 cases per month)	66 (23.2)
More than 1200 cases (about more than 100 cases per month)	39 (13.7)
Number of prescribing suggestions made to physicians in the past year †	
Less than 5 cases	30 (10.5)
5-9 cases (about 1 case in 2 months)	8 (2.8)
10-19 cases (about 1 case per month)	24 (8.4)
20-39 cases (about 2-3 cases per month)	44 (15.4)
40-59 cases (about 4-6 cases per month)	50 (17.5)
60-99 cases (about 7-8 cases per month)	36 (12.6)
More than 100 cases (about more than 8 cases per month)	93 (32.6)
Number of PREAVOID cases in the past year †	
Less than 5 cases	103 (36.1)
5-9 cases (about 1 case in 2 months)	45 (15.8)
10-19 cases (about 1 case per month)	57 (20.0)
20-39 cases (about 2-3 cases per month)	40 (14.0)
40-59 cases (about 5 cases per month)	15 (5.3)
60-99 cases (about 7-8 cases per month)	8 (2.8)
More than 100 cases (about more than 8 cases per month)	17 (6.0)
Number of research presentations at academic conferences †	
No experience	107 (37.6)
1 case	63 (22.1)
2-3 cases	59 (20.7)
4-10 cases	38 (13.3)
More than 10 cases	18 (6.3)
Number of published academic papers †	
No experience	242 (84.9)
1 case	25 (8.8)
2-3 cases	13 (4.5)
4-10 cases	3 (1.1)
More than 10 cases	2 (0.7)

†n (%)

*Median (interquartile range)

### Data and reliability of the psychological scale

The data and Cronbach’s alpha values for each psychological scale are presented in Table 2. The median Resilience (Overall) score was 70/105 (66.7%), the median Innate Resilience score was 39/60 (65.0%), and the median Acquired Resilience score was 31/45 (68.9%). The median self-efficacy score was 71/115 (61.7%) and the median burnout score was 48/75 (64.0%). No ceiling or floor effects were observed for any of the scales.

**Table 2:** Scores and reliability coefficients for each psychological scale

	Median score*	Cronbach’s α
Resilience (overall)	70 (63–77)	0.900
Innate Resilience†	39 (34–43)	0.864
Optimism	10 (8–13)	0.808
Control	10 (8–13)	0.592
Sociability	9 (6–13)	0.880
Vitality	11 (9–13.5)	0.810
Acquired Resilience†	31 (28–36)	0.802
Attempting to solve a problem	10 (9–12)	0.639
Self-understanding	11 (10–12)	0.602
Understanding others	10 (9–12)	0.703
Self-efficacy	71 (64–79)	0.881
Burnout	48 (42–55)	0.856

†Existing factor structure scores created by Hirano

*Median (interquartile range)

### Exploratory factor analysis

Exploratory factor analysis was conducted to confirm the pharmacists’ factor structure for the existing resilience questionnaire. Maximum likelihood and Promax rotation factor analyses were conducted, and five factor structures were identified. The naming of the new factor structure was based on the existing factor structure. Factor 1 was named “A. Perseverance” because the items of perseverance, perseverance to the end, and perseverance in dealing with hard things showed high loadings. Factors 2 and 5 were named “B. Sociability” and “E. Problem-Solving Orientation,” respectively, because the same questions as those in the existing factor structure were extracted. Factor 3 was named “C. Optimism” because, as with the existing factor structure, the optimistic items loaded highly. Factor 4 was named “D. Self-Understanding and Understanding of Others” because the existing items “Understanding of Others’ Psychology” and “Self-Understanding” indicated high loadings. Factors 1 through 3 were interpreted as Innate Resilience and factors 4 and 5 as Acquired Resilience, and the two-dimensional structure was maintained, as in the existing structure.

The details of the new factor structure and reliability coefficient values are presented in Table 3. The cumulative contribution ratio was 60.9% and included items with factor loadings of less than 0.4.

**Table 3:** Exploratory factor analysis

**Resilience (overall), α =.900**	1	2	3	4	5
**Innate Resilience, α = .864**					
A. Perseverance, α = .839					
12. I consider myself a tenacious person.	.924	−.016	−.058	−.088	−.042
8. I can finish what I decide to do.	.846	−.040	.001	−.048	−.053
6. I am a person who values hard work.	.723	.083	−.091	−.092	.055
9. I think I can somehow get through difficult events that come my way.	.485	−.048	.473	.093	−.066
7. I am a person who can endure even the hardest things.	.483	−.149	.186	.134	−.005
13. I treat others with compassion.	.352	.093	−.199	.287	.092
B. Sociability, α = .880					
4. I am good at getting to know people on my own.	−.011	.936	.040	−.041	−.002
2. I have always been good at relating to others.	−.104	.831	−.008	.151	−.057
10. I have a wide range of friendships and I am a good company man.	.085	.759	.051	−.114	.047
C. Optimism, α = .688					
1. I feel that I can usually manage anything.	−.167	−.010	.935	−.051	.137
3. Even if I am not sure about something, I think I can manage it in the end.	.075	.110	.681	.060	−.135
5. I am physically strong.	.149	.133	.228	−.003	.144
**Acquired Resilience, α = .793**					
D. Self-understanding and understanding of others, α = .738					
21. I am relatively good at understanding how others think.	.051	.071	−.111	.698	−.021
17. I am good at reading people's feelings and subtle changes in facial expressions.	.071	.096	−.155	.632	.023
14. I understand my own personality well.	−.103	−.042	.172	.601	−.051
19. I understand how a bad event can affect my feelings.	−.028	−.211	.014	.564	.197
16. I am often unsure of my thoughts and feelings. (*)	.160	−.070	−.155	−.509	.092
11. I can control my emotions even when I have a bad day.	.157	.023	.177	.289	−.008
E. Problem-solving oriented, α = .639					
20. When I have a bad event, I gather information to solve the problem.	−.049	−.035	.007	−.007	.877
18. I actively try to talk to people when I have misunderstandings with them.	.092	.158	.090	.061	.369
15. When I have a bad event, I look for what I can gain from the experience.	.232	.066	.043	.073	.275
Maximum Likelihood Method, Promax Rotation, Cumulative contribution ratio 60.9					

α = Cronbach's α

(*) Reverse-scored items

### Relationships between resilience and related factors

Resilience (overall), Innate Resilience, and Acquired Resilience were all significantly positively correlated with self-efficacy (ρ = 0.743, 0.744, 0.579), significantly negatively correlated with burnout (ρ = −0.436, − 0.439, −0.331), and significantly and weakly positively correlated with percentage of RTOW (ρ = 0.244, 0.230, 0.215). Innate Resilience was significantly and weakly positively correlated with the number of research presentations at academic conferences (ρ = 0.204). No correlations were found for other factors. The details are presented in Table 4.

**Table 4:** Correlations between resilience and related factors

		Resilience (overall)		Innate Resilience		Acquired Resilience
Self-efficacy		.743**		.744**		.579**
Burnout		−.436**		−.439**		−.331**
Age		.029		.033		.003
Years of pharmacist experience		.020		.035		−.008
Years of hospital experience		.019		.036		−.013
Percentage of work						
CPW		.018		.039		−.026
DRW		−.157**		−.172**		−.081
MW		.159**		.147*		.139*
RTOW		.244**		.230**		.215**

The correlation coefficient (ρ) of .20 ≦ | ρ | <.40 is weakly correlated, .40 ≦ | ρ | < .70 is correlated, and .70 ≦ | ρ | ≦ 1.00 is strongly correlated. Tables are expressed in ρ, and only items with significant correlations are shown in bold

Significance level *P<.05　**P<.01

Comparisons across categorical factors showed no significant differences in resilience scores by gender. Regarding work-related activities, overall resilience was significantly associated with the number of patient guidance sessions on medications (*P* =.049, *η*^*2*^_*H*_ = 0.024), research presentations (*P* =.001, *η*^*2*^_*H*_ = 0.055), and published academic papers (*P* =.046, *η*^*2*^_*H*_ = 0.020). Innate resilience was significantly associated with patient guidance (*P* =.003, *η*^*2*^_*H*_ = 0.050), research presentations (*P* =.002, *η*^*2*^_*H*_ = 0.048), and published papers (*P* =.049, *η*^*2*^_*H*_ = 0.019), while acquired resilience was significantly associated only with research presentations (*P* =.003, *η*^*2*^_*H*_ = 0.043). No significant associations were found for the number of prescribing suggestions or PREAVOID cases. The detailed *P*-values and their corresponding effect sizes are shown in Table 5.

**Table 5:** Comparisons between resilience and related factors

	Groups	Resilience (overall)	Innate Resilience	Acquired Resilience
Gender ^1)^	2	*P* = .297, *r* = −.062	*P* = .431, *r* = −.047	*P* = .400, *r* = −.050
Number of patient guidance sessions on medications ^2)^	7	*P* = .049*, *η*^2^_H_=.024	*P* = .003**, *η*^2^_H_= .050	*P* = .619, *η*^2^_H_＜ .01
Number of prescribing suggestions made to physicians ^2)^	7	*P* = .619,* η*^2^_H_＜ .01	*P* = .454, *η*^2^_H_＜ .01	*P* = .583, *η*^2^_H_＜ .01
Number of PREAVOID cases ^2)^	7	*P* = .775, *η*^2^_H_＜ .01	*P* = .590, *η*^2^_H_＜ .01	*P* = .903, *η*^2^_H_＜ .01
Number of research presentations at academic conferences ^2)^	5	*P* = .001**, *η*^2^_H_= .055	*P* = .002**, *η*^2^_H_= .048	*P* = .003**, *η*^2^_H_= .043
Number of published academic papers ^2)^	5	*P* = .046*, *η*^2^_H_= .020	*P* = .049*, *η*^2^_H_= .019	*P* = .148, *η*^2^_H_= .013

Tables are expressed in *P*-value and effect size. Only items with a statistically significant *P*-value are shown in bold.1) Mann–Whitney *U* test, 2) Kruskal-Wallis test

Significance level *P<.05　**P<.01

### Multiple regression analysis

Multiple regression analysis was conducted using a stepwise method with Resilience (overall), Innate Resilience, and Acquired Resilience as objective variables, and each item as an explanatory variable. Self-efficacy and burnout were included for criterion-related validity, as they are known to strongly influence resilience. To explore factors unique to pharmacists, variables with weaker correlations were also deliberately included in the analysis. Although other modeling approaches were considered, the present model was adopted because it yielded the lowest Akaike’s Information Criterion (AIC), achieving an optimal balance between model simplicity and goodness of fit.

For Resilience (overall), four explanatory variables were extracted: self-efficacy, percentage of RTOW, burnout, and gender, with a multiple correlation coefficient (R) of 0.764 and an adjusted R^2^ of 0.577. For Innate Resilience, four explanatory variables were extracted: self-efficacy, burnout, percentage of RTOW, and percentage of CPW, with an R of 0.775 and an adjusted R^2^ of 0.595. For Acquired Resilience, two explanatory variables were extracted: self-efficacy and percentage of RTOW, with an R of 0.594 and an adjusted R^2^ of 0.348.

Self-efficacy was suggested to be the factor most strongly associated with Resilience (overall) (β 0.683, T-value: 15.578), Innate Resilience (β 0.701, T-value: 16.336), and Acquired Resilience (β 0.575, T-value: 11.947). The details are presented in Table 6.

**Table 6:** Multiple linear regression analysis

Resilience (overall)			Model summary: *R*^2^ = .583, Adjusted *R*^2^ = .577, AIC = 1153.8			
	*B*	*SE*	*β*	*T*-value	*P*-value	VIF
(Constant)	34.219	4.924	−	6.950	＜.01**	−
Self-efficacy	0.627	0.040	.683	15.578	＜.01**	1.290
Percentage of RTOW	0.137	0.046	.119	2.981	＜.01**	1.064
Burnout	−0.139	0.050	−.122	−2.757	＜.01**	1.317
Gender (1=F)	−0.099	0.048	−.084	−2.083	.038*	1.080

Innate Resilience			Model summary: *R*^2^ = .601, Adjusted *R*^2^ = .595, AIC = 882.1			
	*B*	*SE*	*β*	*T*-value	*P*-value	VIF
(Constant)	12.951	2.930	−	4.420	＜.01**	−
Self-efficacy	0.408	0.025	.701	16.336	＜.01**	1.294
Burnout	−0.080	0.031	−.111	−2.591	.010*	1.294
Percentage of RTOW	0.078	0.029	.106	2.708	＜.01**	1.082
Percentage of CPW	0.021	0.010	.078	1.990	.048*	1.074

Acquired Resilience			Model summary: *R*^2^ = .353, Adjusted *R*^2^ = .348, AIC = 822.1			
	*B*	*SE*	*β*	*T*-value	*P*-value	VIF
(Constant)	13.029	1.437	−	9.066	＜.01**	−
Self-efficacy	0.238	0.020	.575	11.947	＜.01**	1.009
Percentage of RTOW	0.055	0.025	.105	2.177	.030*	1.009

*R*^2^ Coefficient of determination, *AIC* Akaike’s Information Criterion, *B* Unstandardized regression coefficient, *SE *Standard error, *β *Standardized regression coefficient, *VIF* Variance Inflation Factor

Significance level *P<.05**P<.01

## Discussion

This study revealed the resilience status of clinical pharmacists in Japan. Although previous studies have reported positive correlations between resilience and self-efficacy and negative correlations between resilience and burnout among healthcare professionals [5, 8, 11], our study targeting clinical pharmacists found similar results. In particular, consistent with previous findings, self-efficacy showed the strongest association with resilience, while burnout was negatively associated with it. Additionally, this study is the first to suggest a potential relationship between resilience and the percentage of RTOW, highlighting a factor specific to the Japanese clinical pharmacist population.

The BRS was previously tested for reliability and validity with nurses [6]. The present study verified that the BRS (entire scale and two-dimensional structure) was highly reliable for use with pharmacists. Seven subscales were open for consideration. Compared to the results for nurses, Kasahara et al. [6] reported similar scores, suggesting that the survey data adequately reflected the two-dimensional resilience of pharmacists. The same high reliability was verified for self-efficacy and burnout. We believe that the results adequately reflect the actual conditions of pharmacists.

The results of the validation factorial analysis confirmed the unique five-factor structure of pharmacists. The same two-dimensional structure was confirmed. However, items with low factor loadings were also included, with a cumulative contribution rate of 60.9%. All items were retained in the analysis to preserve the two-dimensional structure and explore potential underlying patterns. This decision was based on the exploratory nature of the study and the theoretical importance of the retained items. There is ample room for the consideration of a pharmacist-specific measure of resilience.

Regarding the relationship with resilience, there were significant correlations with self-efficacy, burnout, and percentage of RTOW. While previous studies have reported associations with self-efficacy and burnout [15], this study identified a weak but clear association with RTOW, which is often performed in addition to normal work. It is assumed that the motivation to gain experience in conference presentations, in addition to the usual work, was associated with Innate Resilience, which was originally considered challenging to acquire.

Multiple regression analysis indicated that self-efficacy was strongly associated Resilience (overall), Innate Resilience, and Acquired Resilience. Unlike the study by Weiss et al., which did not assess self-efficacy [11], the present study considered the characteristics of Japanese pharmacists. For Acquired Resilience in particular, enhancing self-efficacy through regular clinical work, as well as challenges such as research and teaching, may be crucial. Sakai et al. reported that self-efficacy increases with years of experience, but it is also important to assign roles according to abilities, acknowledge successful experiences, and communicate supportive intentions [16]. Clinical experience, reflection, and feedback may therefore promote the development of self-efficacy and, in turn, improve both Acquired and Innate Resilience.

In addition to self-efficacy, engagement in research, teaching, and other work (RTOW) emerged as an important factor associated with Resilience, particularly Acquired Resilience. Although the median proportion of RTOW was only 5%, indicating that these activities are typically undertaken alongside routine pharmacy duties, such engagement may contribute to developing resilience. The combination of primary responsibilities, such as dispensing and patient counseling, with additional professional tasks appears to provide opportunities to cultivate problem-solving skills, adaptive coping, and perseverance. While direct evidence linking RTOW to resilience is limited, repeated challenges and successful task completion in these activities likely enhance self-efficacy, which in turn supports both Acquired and Innate Resilience. Educational activities have also been reported to foster flexible coping, interpersonal and relationship-building skills, help-seeking behavior, stress management, self-confidence, and emotional regulation [17]. Collectively, these findings suggest that engagement in RTOW, alongside routine clinical work, may reinforce resilience and self-efficacy in pharmacists.

The present study also confirmed that burnout is strongly associated with Innate Resilience. While this section focuses primarily on self-efficacy and RTOW, the role of burnout will be discussed in detail in the following section. Patel et al. reported that “emotional exhaustion was found to be the leading contributor of burnout in community pharmacists,” highlighting the need for future research to identify optimal strategies for preventing burnout and promoting resilience in this profession [11, 18]. Understanding individual personalities and characteristics, as well as preventing emotional exhaustion, may play a critical role in maintaining and enhancing Innate Resilience.

### Study limitations

Despite its strengths, this study has some limitations. First, it only investigated pharmacists’ clinical competence in terms of their workload. Although it is necessary to evaluate individual performance in addition to workload to assess professional performance, it is difficult to do so when conducting research. Therefore, in this study, workload was treated as a measure of individual performance. In addition, because this study focused on clinical pharmacists in the hospital setting, caution should be exercised when applying these results to clinical pharmacists working outside hospitals.

Second, the pharmacists in 28 of the 38 facilities participating in this study worked at hospitals affiliated with the same healthcare group. Furthermore, the questionnaire was distributed in specific regions of Japan, which may have resulted in selection bias due to geographical limitations. These factors may have influenced the participants’ responses.

Third, the questionnaire was web-based, and requests for participation were sent to facility directors via email. Although the number of facilities requested could be determined, the number of questionnaires distributed to potential participants was left to the discretion of each facility’s director. Therefore, it was difficult to determine the specific number of people who received the questionnaire or who refused to respond. The inability to calculate the response rate is a limitation of this study.

Fourth, the results of the exploratory factor analysis included points with low cumulative contributions and factor loadings. As mentioned above, the purpose of this study was exploratory, and the creation of a scale is an issue for future research. Therefore, caution should be exercised when interpreting the factor analysis results.

Finally, since this study could not address questions such as “Does engagement in RTOW enhance resilience?” and “If so, is this effect mediated by increased self-efficacy or other factors?” they remain important topics for future research. Moreover, it was not possible to determine whether unmeasured factors acted as confounders. Future research will examine the structural relationships among unmeasured variables related to resilience, including their potential roles as confounders or mediators. In addition, the research team aims to develop a resilience scale specific to pharmacists that not only demonstrates strong explanatory power but also shows good model fit for both clinical and research applications.

## Conclusions

This study revealed the relationship between resilience, self-efficacy, and RTOW among clinical pharmacists in Japan. Criterion-related validity was also evidenced by high self-efficacy. The novel identification of RTOW as an associated factor in this context provides insights for further development of the scale. As the stress and problems faced by pharmacists differ depending on the type of work, the development of a resilience scale unique to pharmacists is needed in the future.

## Data Availability

The datasets used and/or analyzed in this study are available from the corresponding author upon reasonable request.

## Abbreviations

PREAVOID: PREvent and AVOID adverse drug reactions
CPW: Clinical Practice Work
DRW: Dispensing Room Work
MW: Managerial Work
RTOW: Research, Teaching, and Other Work
BRS: Bidimensional Resilience Scale
JGSES: Japanese version of the Generalized Self-Efficacy Scale
JBS: Japanese Burnout Scale

## References

[1] Ministry of Health, Labour and Welfare. Outline of the model core curriculum for pharmaceutical education (Revised 2022).https://www.mhlw.go.jp/content/11121000/001079344.pdf (2022). Accessed 31 Oct 2024.

[2] Ministry of Health, Labour and Welfare. Clinical training guidelines for pharmacists. https://www.mhlw.go.jp/content/001234125.pdf (2024). Accessed 31 Oct 2024.

[3] Garcia-Dia MJ, DiNapoli JM, Garcia-Ona L, Jakubowski R, O’Flaherty D. Concept analysis: resilience. Arch Psychiatr Nurs. 2013;27:264–70. 10.1016/j.apnu.2013.07.003. 24238005 10.1016/j.apnu.2013.07.003

[4] Sunami N. Resilience in clinical nurses: a concept analysis. J St Lukes Soc Nurs Res. 2018;22:13–20. 10.34414/00015302.

[5] Negi K, Katayama H. Impact of self-esteem and self-efficacy on resilience among female senior clinical nurses. J Jpn Acad Nurs Sci. 2018;38:89–96. 10.5630/jans.38.89.

[6] Kasahara S, Sugimoto C, Oka K. Validating the bidimensional resilience scale among nursing students and nurses. J Jpn Acad Nurs Sci. 2018;38:160–8. 10.5630/jans.38.160.

[7] Hirano M. A study of the classification of resilience factors: development of the bidimensional resilience scale (BRS). Jpn J Personal. 2010;19:94–106. 10.2132/personality.19.94.

[8] Tomita K, Hanaki K. Resilience and its related factors among new nurses at university hospitals. J Yonago Med Ass. 2021;72:26–33. https://repository.lib.tottori-u.ac.jp/records/4309.

[9] Saseen JJ, Ripley TL, Bondi D, Burke JM, Cohen LJ, McBane S, et al. ACCP clinical pharmacist competencies. Pharmacotherapy. 2017;37:630–6. 10.1002/phar.1923. 28464300 10.1002/phar.1923

[10] Schommer JC, Gaither CA, Goode JR, Owen JA, Scime GM, Skelton JB, et al. Pharmacist and student pharmacist views of professional and personal well-being and resilience. J Am Pharm Assoc. 2020;60:47–56. 10.1016/j.japh.2019.09.006. 10.1016/j.japh.2019.09.006 31669419

[11] Weiss SS, Weiss L, Clayton R, Ruble MJ, Cole JD. The relationship between pharmacist resilience, burnout, and job performance. J Pharm Pract. 2024;37:644–9. 10.1177/08971900231164886. 36938593 10.1177/08971900231164886

[12] Hirano M. Can resilience be acquired? Understanding and supporting individual differences. Tokyo: University of Tokyo; 2015.

[13] Narita K, Shimonaka Y, Nakazato K, Kawaai C, Sato S, Osada Y. A Japanese version of the generalized Self-Efficacy scale: scale utility from the life-span perspective. Jpn J Educ Psychol. 1995;43:306–14. 10.5926/jjep1953.43.3_306.

[14] Bandura A. Self-efficacy: toward a unifying theory of behavioral change. Psychol Rev. 1977;84:191–215. 10.1037/0033-295X.84.2.191. 847061 10.1037//0033-295x.84.2.191

[15] Kubo M. The factorial and construct validity of the Japanese burnout scale among service workers. Jpn J Psychol. 2014;85:364–72. 10.4992/jjpsy.85.13214. 10.4992/jjpsy.85.13214 25486843

[16] Sakai T, Togashi-Arakawa C. Factors influencing self-efficacy in mid-career nurses. J Jpn Health Med Assoc. 2017;26:65–73. 10.20685/kenkouigaku.26.2_65.

[17] Fukushima M, Miyashita T. Examining factors that increase teacher resilience—towards developing resilience in teacher training courses. Bull Joetsu Univ Educ. 2024;44:1–17. https://juen.repo.nii.ac.jp/record/2000290/files/25.pdf.

[18] Patel SK, Kelm MJ, Bush PW, Lee HJ, Ball AM. Prevalence and risk factors of burnout in community pharmacists. J Am Pharm Assoc. 2021;61:145–50. 10.1016/j.japh.2020.09.022. 10.1016/j.japh.2020.09.022 33069594

